# Effect of Transplantation of Bone Marrow Derived Mesenchymal Stem Cells and Platelets Rich Plasma on Experimental Model of Radiation Induced Oral Mucosal Injury in Albino Rats

**DOI:** 10.1155/2017/8634540

**Published:** 2017-02-26

**Authors:** Basma Elsaadany, Samar El Kholy, Dalia El Rouby, Laila Rashed, Tarek Shouman

**Affiliations:** ^1^Oral Medicine, Oral Diagnosis and Periodontology Department, Faculty of Dentistry, Cairo University, Giza, Egypt; ^2^Oral Pathology, Faculty of Dentistry, Cairo University, Giza, Egypt; ^3^Biochemistry Department, Faculty of Medicine, Cairo University, Giza, Egypt; ^4^Radiation Oncology, National Cancer Institute, Cairo University, Giza, Egypt

## Abstract

Normal tissue damage following radiotherapy is still a major problem in cancer treatment. Therefore, the current work aimed at exploring the possible role of systemically injected bone marrow derived mesenchymal stem cells (BM-MSCs) and/or locally injected platelet rich plasma (PRP) in ameliorating the side effects of ionizing radiation on the rat's tongue. Twelve rats served as control group (N) and 48 rats received a single radiation dose of 13 Gy to the head and neck region; then, they were equally divided into 4 experimental groups: irradiated only (C), irradiated + MSCs (S), irradiated + (PRP) (P), and combined group (PS). Animal scarification occurred in 3 and 7 days after radiation. Then, tongues were dissected and examined histologically and for expression of bcl-2 by RT-PCR. Histological examination of the treated groups (S), (P), and (PS) revealed an obvious improvement in the histological structure of the tongue, compared to group (C), in addition to upregulated expression of bcl-2, indicating decreased apoptotic activity.* Conclusion*. BM-MSCs and PRP have shown positive effect in minimizing the epithelial atrophy of normal oral mucosa after regional radiotherapy, which was emphasized by decreasing apoptotic activity in these tissues. Nevertheless, combined use of BM-MSCs and PRP did not reveal the assumed synergetic effect in oral tissue protection.

## 1. Introduction

Mostly one hundred percent of those experiencing high dose radiation therapy for head and neck cancers suffer from some degree of oral mucositis [1, 2]. Chemotherapy or radiotherapy may initiate mucositis directly by causing DNA strand breaks, through the generation of reactive oxygen species (ROS), or through enzymatic or transcription factor activation in multiple cellular elements within the mucosa [3]. This complex mechanism ends by diminished regenerative capacity of the oral and alimentary epithelium, leading to atrophy, erythema, ulceration, and, usually, the loss of mucosal barrier [4]. However, most of existing interventions for oral mucositis are only palliative, neither specific nor efficient at preventing or treating this complication. Several studies have reported the use of mesenchymal stromal cells derived from bone marrow (BM-MSCs) to manage oral mucosal lesions, and this method is now being used to treat many clinical cases [5] Stem cells are the precursors of the body tissue, defined as immature or undifferentiated cells that are capable of generating daughter cells identical to themselves or of differentiating into diverse cellular phenotypes [6]. The use of stem cells in oral ulcers and wound healing is based on good integration of cell migration and proliferation, extracellular matrix deposition, angiogenesis, and remodeling. BM-MSCs are self-renewing, expandable stem which possess the ability to engraft at the site of injury and promote tissue regeneration and wound healing through synergistic down regulation of proinflammatory cytokines and increased production of soluble factors with antioxidant, antiapoptotic, and proangiogenic properties [7].

In addition, mucosal healing with PRP was also reported, and PRPs advanced the angiogenic response of oral mucosal wounds during the first 10 days following surgery [8]. During the healing process, cells can be attracted to the wound position mainly because of local release of growth factors and endogenous signals contributing to a fast wound healing [9, 10] In vitro studies reveal that PRP can be a potent tool for attraction of cell populations, such as MSCs at the wound area [11]. Consequently, the present preclinical study was established to assess the preventive/regenerative capacity of BM-MSCs, PRP, and their combination for radiation induced oral mucositis in terms of preserving the epithelium integrity and decreasing the apoptotic activity.

## 2. Materials and Methods

### 2.1. Experimental Animals and Ethical Statement

The present study was conducted on 60 healthy male albino rats, age ranged between 3 and 4 months, and weight was about 100 to 150 grams. The sample size of the present study was divided into 12 rats in each group, which were further divided into 2 blocks (6 animals in each block). This was satisfactory to achieve normality of data as no previous similar research was done (with the same comparison groups) on this particular disease and interventions. The animals were obtained and housed under standardized conditions with controlled temperature and humidity (30–35%) and a 12-12 h light-dark cycle.

The rats had free access to standard rat chow and tap water at the Research Animal House of National Cancer Institute, Cairo, Egypt.

All aspects of the animals care and experimental protocols were reviewed and approved by ethical committee in the Faculty of Oral and Dental Medicine, Cairo University. Furthermore, the protocol was in accordance with the recommendations of the National Institutes of Health guide for the care and use of laboratory animals (NIH Publications No. 8023, revised 1978).

### 2.2. Experimental Design

The selected rats were randomized into five groups, one normal control group and four experimental groups. In experimental groups, rats were anaesthetized and then received a single radiation dose of 13 Gray (Gy) to the head and neck region; then they were equally subdivided according to intervention used into four groups: irradiated only group (C), irradiated + mesenchymal stem cells (MSCs) group (S), irradiated + platelet-rich plasma (PRP) group (P), and combined group (PS). The animals in each group were further divided into two blocks as scarification occurred in 3 and 7 days after radiation. Then, after scarification, the tongue were dissected to a specimen of 5 mm in diameter, which was taken from the lateral border of right side of the tongue and kept frozen in −7°C for RT- PCR. The remainder of the tongue tissue was fixed in 10% formalin as a step in specimen preparation for histological examination. Epithelial thickness and number of blood vessels were assessed histologically in H&E section, and apoptotic activity was assessed through RT–PCR for bcl-2.

### 2.3. Experimental Procedure

#### 2.3.1. Rat Anesthesia

The rats were anaesthetized by i.p. injection of sodium pentobarbital (Nembutal®, 40 mg/kg body weight) and ketamine chloride (ketalar®, 40 mg/kg body weight).

#### 2.3.2. Radiation Exposure

Irradiation of the anesthetized rats was performed at the National Cancer Institute, Cairo, Egypt, using linear accelerator (Electa-precise T System) using 4 MeV electron beam with 1 cm bolus. The field portal was customized by lead cutout. Heads of five rats were placed in the same irradiation field during radiation exposure. Thirteen Gray of radiation was delivered at a single dose to all of the experimental rats. The source of radiation, the dose, and the scarification dates were determined based on a pilot study to generate mucositis model in rats in response to regional radiation using the same method of irradiation described before in the present experiment with different doses. Survival rate and histologic changes, mainly epithelial thickness of keratinized mucosa, were evaluated.

#### 2.3.3. Treatment Protocol

Mesenchymal stem cells (MSCs) group (S) received injection with 1 × 10^7^ cells in 0.2 mL labeled with PKH26 fluorescent linker dye in phosphate buffer saline (PBS) [12]. The injection was at lateral tail vein immediately following irradiation. Platelet-rich plasma (PRP) group (P) received local injection with PRP in right lateral border of the tongue of irradiating the rats immediately following irradiation. Injection was done using a 1 mL insulin syringe with a needle size 27 gauge × 1/2 inch (BD Nokor™). Combined group (PS) received combination therapy of group (P) and group (S).

#### 2.3.4. Preparation of BM-MSCs

Ten 6-week-old male white Albino rats (100–120 g) were sacrificed by cervical dislocation, and marrow was isolatedat the Research Animal House of National Cancer Institute, Cairo, Egypt. Bone marrow was harvested by flushing the tibiae and femurs of rats with DMEM (GIBCO/BRL) supplemented with 10% fetal bovine medium (GIBCO/BRL). Nucleated cells were isolated with a density gradient [Ficoll/Paque (Pharmacia)] and were resuspended in a complete culture medium supplemented with 1% penicillin-streptomycin (GIBCO/BRL). The cells were then incubated in a CO_2_ incubator at 37°C in 5% humidified CO_2_ for 12–14 days as a primary culture or upon the formation of large colonies. When large colonies developed (80–90% confluence), the cultures were washed twice with phosphate buffer saline (PBS) and the cells were trypsinized with 0.25% trypsin in 1 mM EDTA (GIBCO/BRL) for 5 minutes at 37°C. After centrifugation, cells were resuspended in PBS. MSCs in culture were characterized by their adhesiveness and fusiform shape and by detection of CD29, one of the surface markers for MSCs by RT- PCR [13].

Labeling of stem cells with PKH26 dye is as follows: MSCs cells were harvested during the 4th passage and were labeled with PKH26 fluorescent linker dye. PKH26 is a red fluorochrome having 551 nm excitation and 567 nm emission. The labeled cells retain both their biological and proliferating activities. Thus, the linker is ideal for in vitro cell labeling, in vitro proliferation studies and long term, in vivo cell tracking. The dye itself is stable and divides equally when the cells divide. Detection of homing of the injected cells in the rat's tongue is as follows: the rat's tongue was examined with a fluorescent microscope to detect the cells stained with PKH26 dye to ensure engraftment of the injected cells into the irradiated tongue.

#### 2.3.5. Protocol for PRP Preparation

**Figure 1:**
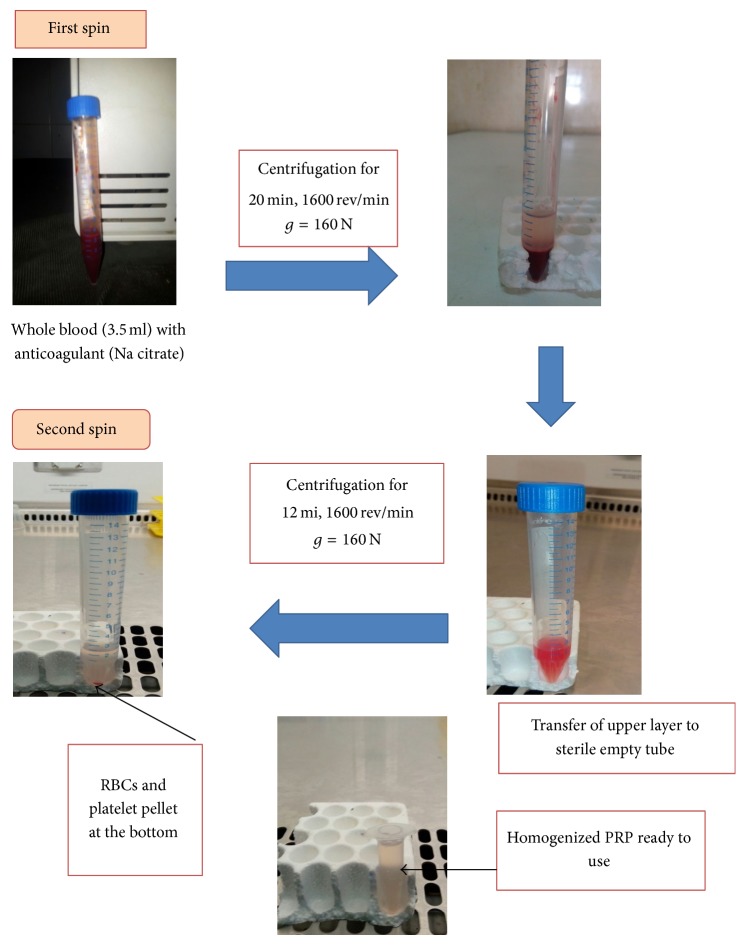
Flowchart describing preparation of PRP.

A 3.5 mL volume of autologous blood was drawn from 24 animals into vacuum tubes containing 10% sodium citrate. The PRP was prepared according to a double-centrifugation protocol [14] (Figure 1).

#### 2.3.6. Specimen Preparation

All animals were sacrificed by cervical dislocation at 3 and 7 days following irradiation; the tongue was dissected out and fixed in 10% neutral buffered formalin. Specimens were then dehydrated through graded alcohol, cleared in xylene, and embedded in paraffin. Sections of 4–6 *μ* thickness were prepared and subjected to haematoxylin and eosin staining for routine histological examination.

#### 2.3.7. Histological Evaluation

The assessor was completely blind about the sample groups during evaluation. The thickness of epithelial lining of the dorsal surface of the tongue was estimated using Leica Quin 500 analyzer computer system (Leica Microsystems, Switzerland). The cursor was used to draw a straight line representing the distance from the basement membrane to the upper most layer of epithelial cells in H&E stained sections. The image analyzer is calibrated automatically to convert the measurement units (pixels) produced by the image analyzer program into actual micrometer units. The thickness was estimated in 2 different points, in 2 different fields, in each specimen using magnification (×100). Mean values and standard deviation (SD) were calculated for each group. The number of blood vessels was counted using a magnification of ×100 (measuring frame area = 299762 *μ*m^2^) in 5 measuring fields in each specimen. Finally, mean values were obtained for each case.

#### 2.3.8. QRT-PCR Gene Expression of bcl-2 in Rat Tissues

In brief, total RNA was isolated from biopsy of each rat and purified using the (bax∣bcl-2) kit (Qiagene, USA) according to the manufacturer's protocol. Total RNA was then converted into cDNA using specific miRNA primer probes, for (bax∣bcl-2). The RT–PCR product was diluted 10-fold and mixed with Universal PCR Master Mix (TaqMan, Applied Biosystem, USA). miRNA expression analysis was performed using QRT-PCR, according to the manufacturer's protocols (Applied Biosystems).

#### 2.3.9. Statistical Analysis

Statistical analysis was done using Statistical Package for Social Sciences (SPSS), Version 18.0 for Windows. Continuous variables were analyzed as mean values ± standard deviation (SD). As the data following normal distribution, the analysis of variance (ANOVA) test was used to compare the mean of the variables (epithelial thickness, number of blood vessels, and bcl-2 expression) between the experimental and control groups in different scarification dates, followed by post hoc Tukey test, if results of (ANOVA) test was significant. *p* value of ≤.05 was considered statistically significant.

## 3. Results

### 3.1. Histological Results

#### 3.1.1. Irradiated Only Group

**Figure 2:**
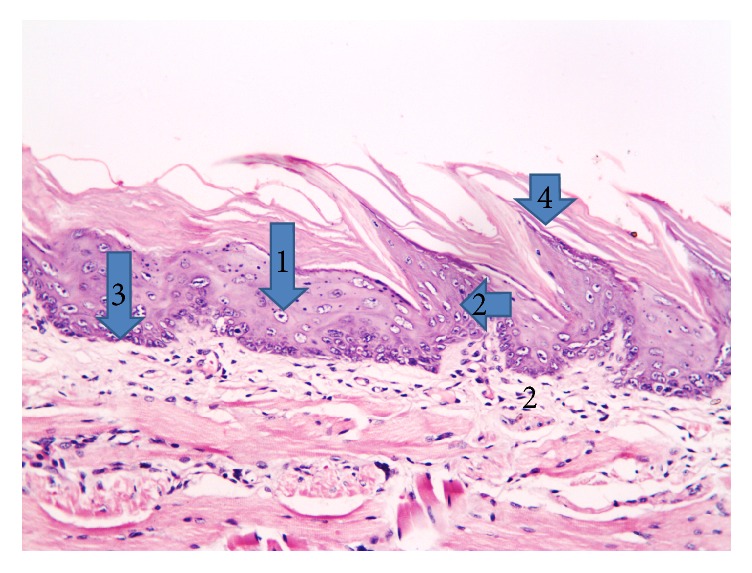
Photomicrograph of the dorsal surface of the tongue of the irradiated only group at 3 days following radiation showing (1) cytoplasmic perinuclear vacuolization, (2) apoptotic cells, (3) disturbed architecture of basal cells, and (4) keratin tips that were lost over some papillae (H&E, ×200).

Three days following irradiation, crowded deeply stained nuclei and disrupted architecture of basal cells were noted. Keratin tips were lost over some papillae. In the underlying lamina propria, some blood vessels were dilated; areas of edema and mild chronic inflammatory cell infiltrate could also be noticed (Figure 2).

**Figure 3:**
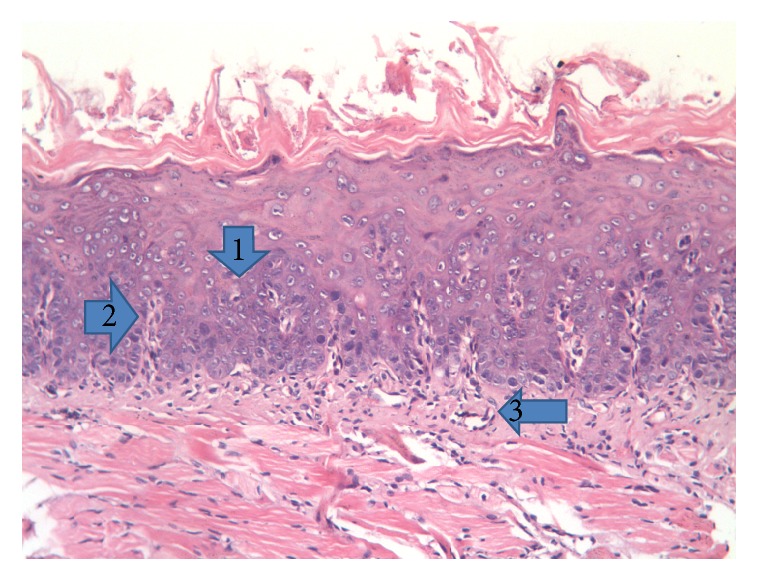
Photomicrograph of the dorsal surface of the tongue of the irradiated only group at 7 days following radiation showing (1) cytoplasmic perinuclear vacuolization, (2) normal architecture of basal cells, and (3) small blood vessels (H&E, ×200).

Seven days following irradiation, the dorsal surface of the tongue presented nearly normal keratinized stratified squamous epithelium without obvious changes in its structure except for localized areas showing reduced keratin height over some lingual papillae, in comparison with control group. The lamina propria beneath the covering epithelium sometimes showed some dilated blood vessels containing RBCs, together with areas of degeneration and edema (Figure 3).

#### 3.1.2. Treated Groups (Irradiated and MSCs and/or PRP Treated Group)

**Figure 4:**
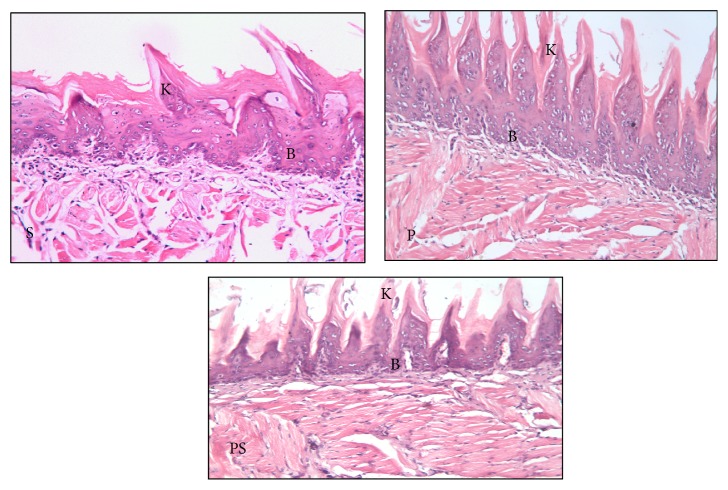
Photomicrographs of the dorsal surface of the tongue of the treated groups [(S) irradiation + MSCs, (P) irradiation + PRP, (PS) irradiation + MSCs + PRP] at 3 days following radiation showing slight alteration in basal cell architecture (B) and preserved keratin tips over the lingual papillae (K) (H&E, ×200).

*Three Days following Irradiation*. The dorsal surface of the tongue showed preserved keratin tips over most lingual papillae mainly in PRP groups. Besides, cellular changes could be noticed among the cells of keratinized stratified squamous epithelium in the form of few intracytoplasmic vacuoles, when compared to those in the irradiated group (Figure 4).

Altered basal cell architecture was still noticed but was less obvious in comparison to the irradiated only group. In the underlying lamina propria, smaller areas of degeneration and edema among the collagen fibers were observed (Figure 4). 

**Figure 5:**
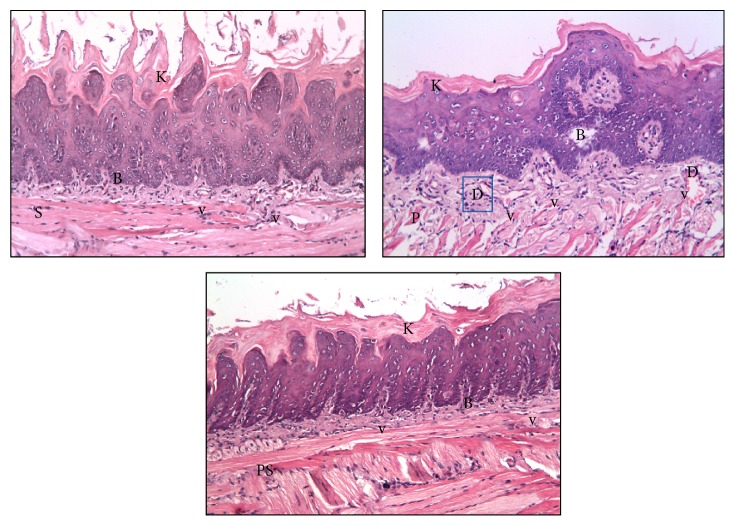
Photomicrographs of the dorsal surface of the tongue of the treated groups [(S) irradiation + MSCs, (P) irradiation + PRP, (PS) irradiation + MSCs + PRP] at 7 days following radiation showing (B) preserved basal cell architecture, (K) preserved keratin tips over the lingual papillae, (v) blood vessels, and (D) dilated engorged blood vessel (H&E, ×200).

*Seven Days following Irradiation*. The dorsal surface of the tongue revealed no significant change in comparison to the irradiated group at the same time of scarification except that the keratin and lingual papillary height and the basal cell layer architecture were apparently preserved in most areas. Fewer blood vessels were detected in the lamina propria, with a relatively increased number in the PRP treated groups (Figure 5).

At the end point of the study (7 days) the rat's tongue was examined with a fluorescent microscope to detect the cells stained with PKH 26 dye; results obtained insure successful engraftment of systemically injected cells into the irradiated tongue in the S group and PS group.

### 3.2. Epithelial Thickness

After 3 days of irradiation, the radiation caused a significant decrease in epithelial thickness mean value in comparison to normal thickness, while the stem cell and/or PRP attenuated the effect of radiation on epithelial thickness of dorsal surface of rats' tongue, with no intervention being apparently superior than the others. However, the epithelial thickness in treatment groups was still significantly different from normal thickness (Table 1). Seven days after radiation, the epithelial thickness mean value of irradiated only group was still significantly less than both normal control and other intervention groups, while at this date there was no significant difference in epithelial thickness mean values between [stem cell and/or PRP] groups and normal control group (Table 2).

### 3.3. Number of Blood Vessels

At 3 days after radiation, there is an increase in number of blood vessels mean value as a response to radiation compared to normal control and there was no significant effect for stem cell and/or PRP in this time point (Table 1). However, at 7 days after irradiation the immediate local use of PRP yielded a significant increase in number of blood vessels compared to other intervention groups (radiation + stem cell and radiated only group) and normal control (Table 2).

Besides, results also suggested that, compared to the irradiated only group, immediate systemic use of stem cells only had no significant effect on the observed increase in number of blood vessels at 7 days after irradiation.

### 3.4. Expression of bcl-2

At 3 and 7 days after regional radiation, there was a significant increase in apoptosis as a response to radiation compared to normal control and there was a significant effect of stem cell and/or PRP in this time point in decreasing the apoptotic activity (Tables 1 and 2). In addition, the results suggested that at 3 and 7 days after irradiation the effect of immediate local use of PRP in combination with systemic use of stem cell yielded the most significant decrease in apoptosis compared to other intervention groups (radiation + PRP, radiation + stem cell, and radiated only group) almost reaching the normal level at 7 days after irradiation (Tables 1 and 2).

## 4. Discussion

Regarding the animal model for our work, a rat tongue was the model of choice, although previously described models for oral mucositis frequently used mice. It is technically difficult to manipulate mice sampling due to the small size of the typical mouse [15, 16]. Tongue mucositis model was preferred for this study, since it provides the best reproducibility and adequate tissue sampling. Additionally, the dorsal surface of the tongue covered with keratinized epithelium and nonkeratinized epithelium and can express the epithelial tissues of oral mucosa. Our main concern in this study was prevention rather than treatment. We performed a pilot study to determine the least regional dose used that is going to affect the mucosal tissue with 100% survival rate. Most of the previous studies in their models to produce radiation mucositis in mice clinically applied the radiation directly on the tongue or the mouse snout and with larger doses [17, 18] so the brain as a vital organ did not receive high dose of radiation. In our model we were evaluating if using the studied interventions is beneficial in reversing the early histologic changes. Moreover, that also explains why we preferred the dorsal surface although is not commonly affected in clinical practice, since we aimed to evaluate the potential of the studied treatments to prevent radiation induced alteration, not only in nonkeratinized mucosa but also in keratinized mucosa. In the design of our pilot study we began with the technique done by Abushady et al. [12] using regional radiation to the head and neck to study the effect of MSCs on nonkeratinized mucosa. We modified the technique, source of radiation, the dose, and time of evaluation to get the highest dose inducing atrophy in the dorsal surface with no animal loss in the observation period (14 days). Moreover, it was more convenient to choose the scarification date depending on the normal turn over period in the normal tissue, and this enabled us to detect the early changes in the tissue, given that the mean turnover time for dorsal surface of the tongue is 3.2 days [19]. The immediate systemic injection of MSCs was according to [20] who found that the irradiated tissues express certain receptors/ligands in a gradient that facilitates attraction, adhesion, and engraftment of stem cells to the damaged site. Accordingly, stem cell therapy may be a promising therapeutic alternate to improve RT-induced tissue damage.

The obtained histological results about radiation effect were in agreement with those reported by [21] who revealed changes in the nuclear shape, plus increased vacuolization in primary cultures of rainbow trout skin exposed to gamma radiation. The authors suggested that nuclear changes were caused by increased metabolic activity or disturbed mitosis. The reduction in epithelial thickness of the irradiated rat's tongue is in agreement with the result obtained by [22] who reported reduced mucosal thickness in ventral surface of the tongue of mice at 4 days following irradiation. Regarding apoptosis, a decrease in the antiapoptotic protein BCl-2 after receiving radiotherapy is consistent with prior studies in animals, human cell lines, and intestinal crypts after treatment with chemotherapy and/or radiotherapy and is correlated to increased apoptotic activity which results in intestinal mucositis [23–25]. Conversely, we had recorded an upregulation of antiapoptotic proteins BCl-2 with time at 7 days corresponding with improvement of the histological appearance. Remarkably, these findings correlate with the five-phase theoretical mucositis model suggested by Sonis [26].

We reported that MSC are able to restore integrity by maintaining cellular and tissue homeostasis, through the specific regulation of epithelial cells, and reduce radiation-induced epithelial apoptosis in the tongue.

This histologic improvement detected in our study is supporting the recent clinical results of Maria et al. [27] who reported that syngeneic freshly cultured adipose MSCs therapy resulted in a significant 72% reduction in radiation induced oral mucositis duration by increasing the clinically relevant ulceration latency and accelerating its healing. The model of radiation mucositis in this study is similar to our model in being the result of single dose irradiation but with different radiation dose, application technique, and route of cells administration (using the intraperitoneal route). In addition they depended on clinical endpoint in their study Moreover, Maria et al. [27] revealed that MSC dose size and frequency, number of doses, and onset of treatment are the main parameters that were optimized to yield the most favorable therapeutic benefit. Similarly, Schmidt et al. [17] in his single dose model showed that there was a significant improvement of the ED50 of radiation induced oral mucositis with intravenous transplantation of bone marrow and bone marrow derived MSCs (ED50 is the RT dose needed to cause epithelial ulceration in 50% of animals).

In our study, the small number of PKH26 labeled bone marrow derived stem cells implanted into the tongue mucosa means that the replacement of epithelial cell loss by trans-differentiation is unlikely to represent the main therapeutic principle. Nevertheless, the effect of MSC reported in these studies could be the consequence of releases and synergic effects of multiple paracrine factors. Dong and his group have documented MSCs synthesis and release of numerous cytokines and growth factors such as IL-11, HGF, FGF-2, and IGF-I [28]. Each of these factors has been described previously as facilitating mucosal repair, through enhancement of cell proliferation or inhibition of epithelial cell apoptosis, or by a combination of both [29–33]. However, because of the plasticity of stem cells, transplantation with stem/progenitor cells has been reported to activate tumor proliferation, angiogenesis, and metastasis in several studies [34, 35], whereas other studies showed that stem/progenitor cells suppressed tumor activity [35, 36]. In any case, the fact that transplanted BMDCs could not trans-differentiate into tongue epithelial cells leads us to hypothesize that BMDCs have not much risk of contributing to tumor growth.

The effect of PRP found in this study could be explained in the light of many studies, both in vitro and in vivo, that revealed the effectiveness of growth factors derived from platelets to augment cell proliferation, differentiation, chemotaxis, angiogenesis, and extracellular matrix synthesis involved in the healing of mucositis after irradiation [37, 38]. Unique angiogenic character of PRP found in this study could be attributed to the platelet functions, in addition to coagulation factors. Platelets also store and release many bioactive angiogenic factors including platelet-derived epidermal growth factor (PD- EGF), platelet-derived growth factor (PDGF), VEGF, basic fibroblast growth factor (bFGF), angiopoietins (Angs), and transforming growth factor beta (TGF-*β*) [38, 39] making platelets important in angiogenesis [40, 41]. Interestingly, results obtained from this study indicate that the combined use of PRP and MSCs did not enhance the regenerative response as expected. Our data are in agreement with the finding of [42] who found that PRP treatment offers no additional benefit on the effect of MSCs in improving the healing response in sheep after induced wound. Conversely, data presented by [43] about MSCs/PRP admixtures show that MSCs, migrate effectively into and through PRP, which acts as a highly appropriate vehicle for delivering cells by injection to reconstruct bony defects in a clinical setting. This controversy could be attributed to the varied response in different tissues, difference in origin of the stem cells used, method of delivery of the cells, and PRP concentration.

## 5. Conclusion

Using the radiation protocol used in this study, MSCs and PRP were able to prevent or minimize radiation induced mucositis. Further investigations using different radiation doses, number, or frequency are still required for proper demonstration of the advantageous effects of these treatments. Furthermore, additional research is needed to ascertain the source, processing method, and delivery root of MSC that can generate the best treatment outcome. We also recommend further long-term studies to evaluate the safety of MSCs and PRP (e.g., tumor protection, growth stimulation of residual tumor cells).

## Figures and Tables

**Table 1:** Comparison of (epithelial thickness, number of blood vessels, and expression of bcl-2) between control group and different experimental groups after 3 days of irradiation.

Parameter	Normal control	Radiated only	Radiation + MSCs	Radiation + PRP	Radiation + MSCs + PRP	*p* value
(1) Epithelial thickness						
M	108.3^a^	23.63^b^	65.08^c^	77.15^c^	80.40^c^	.0005^*∗*^
SD	5.63	7.06	7.23	16.59	11.51	

(2) No. of blood vessels						
M	5.50^a^	6.14^a^	6.16^a^	9.66^b^	8.83^b^	.0005^*∗*^
SD	.547	1.57	1.47	2.16	2.48	

(3) Expression of bcl-2						
M	1.02^a^	.20^b^	.72^c^	.5^c^	.81^d^	.0005^*∗*^
SD	.024	.062	.077	.010	.105	

^*∗*^Significant difference at (*p* < .05)

(1) Unit *µ*m, (2) unit number, and (3) unit relative expression to control

Means with different superscript letters (a, b, c, and d) within the same row are significantly different.

**Table 2:** Comparison of (epithelial thickness, number of blood vessels, and expression of bcl-2) between control group and different experimental groups at 7 days after irradiation.

Parameter	Normal control	Radiated only	Radiation + MSCs	Radiation + PRP	Radiation + MSCs + PRP	*p* value
(1) Epithelial thickness						
M	104.9^a^	72.45^b^	97.98^a^	87.36^a^	93.86^a^	.0005^*∗*^
SD	9.13	13.06	10.91	17.71	5.640	

(2) No. of blood vessels						
M	5.33^a^	8.85^b^	7.85^b^	11.33^c^	11.42^c^	.0005^*∗*^
SD	1.52	1.34	1.06	3.55	1.27	

(3) Expression of bcl-2						
M	1.01^a^	.188^b^	.88^c^	.72^d^	1.01^a^	.0005^*∗*^
SD	.023	.0640	.079	.045	.094	

^*∗*^Significant difference at (*p* < .05)

(1) Unit *µ*m, (2) unit number, and (3) unit relative expression to control

Means with different superscript letters (a, b, c, and d) within the same row are significantly different.
